# Residual Bone Height and New Bone Formation after Maxillary Sinus Augmentation Procedure Using Biomaterials: A Network Meta-Analysis of Clinical Trials

**DOI:** 10.3390/ma16041376

**Published:** 2023-02-06

**Authors:** Shahnavaz Khijmatgar, Massimo Del Fabbro, Margherita Tumedei, Tiziano Testori, Niccolò Cenzato, Gianluca Martino Tartaglia

**Affiliations:** 1 Department of Biomedical, Surgical and Dental Sciences, University of Milan, 20122 Milan, Italy; 2 Department of Oral Biology and Genomic Studies, AB Shetty Memorial Institute of Dental Sciences, Nitte (Deemed to be University), Mangalore 575018, Karnataka, India; 3 Fondazione IRCCS Ca’ Granda Ospedale Maggiore Policlinico, 20122 Milan, Italy; 4 Department of Implantology and Oral Rehabilitation, Dental Clinic, IRCCS Ospedale Galeazzi-Sant’Ambrogio, 20157 Milan, Italy; 5 Department of Periodontics and Oral Medicine, School of Dentistry, University of Michigan, Ann Arbor, MI 48109, USA

**Keywords:** biomaterials, bone substitutes, maxillary sinus augmentation, network meta-analysis, sinus floor elevation

## Abstract

Background. Different factors may affect new bone formation following maxillary sinus floor augmentation for the rehabilitation of posterior edentulous maxilla. The purpose of this study was to determine the influence of residual bone height (RBH) on new bone formation after lateral sinus augmentation utilizing different biomaterials, through a network meta-analysis (NMA). Methods. PUBMED, Scopus, and Web of Science electronic databases were searched until 31 December 2022 to obtain relevant articles. A hand search was also conducted. Randomised controlled studies on maxillary sinus augmentation comparing different grafting materials in patients with atrophic posterior maxilla, in need of prosthetic rehabilitation, were included. The risk of bias was assessed following the guidelines of the Cochrane Collaboration. The primary outcome was new bone formation (NBF), assessed histomorphometrically. The statistical analysis was performed by splitting the data according to RBH (<4 mm and ≥4 mm). Results. A total of 67 studies were eligible for conducting NMA. Overall, in the included studies, 1955 patients were treated and 2405 sinus augmentation procedures were performed. The biomaterials used were grouped into: autogenous bone (Auto), xenografts (XG), allografts (AG), alloplasts (AP), bioactive agents (Bio), hyaluronic acid (HA), and combinations of these. An inconsistency factor (IF) seen in the entire loop of the XG, AP, and Bio+AP was found to be statistically significant. The highest-ranked biomaterials for the <4 mm RBH outcome were XG+AG, XG+AP, and Auto. Similarly, the surface under the cumulative ranking curve (SUCRA) of biomaterials for ≥4 mm RBH was Auto, Bio+XG, and XG+Auto. Conclusion. There is no grafting biomaterial that is consistently performing better than others. The performance of the materials in terms of NBF may depend on the RBH. While choosing a biomaterial, practitioners should consider both patient-specific aspects and sinus clinical characteristics.

## 1. Introduction

The maxillary sinus augmentation is a popular surgical procedure for the rehabilitation of atrophic posterior maxilla, consisting of the lifting of the sinus floor by the insertion of biomaterials [1,2,3]. It facilitates an increase in bone height for the placement of dental implants. The outcome of this procedure depends upon several factors. The latter include the type of surgery (e.g., lateral or trans-crestal approach), the implant features (e.g., macro- and microgeometry, connection with the abutment), the patient health status (e.g., drugs taken, systemic conditions), the type of grafting material (e.g., autogenous bone or bone substitutes), and local factors, including residual bone quality and quantity [2]. If residual bone height (RBH) is >10 mm, in most cases there is no need to undergo the sinus augmentation procedure. If the height is between 7–10 mm, a trans-crestal sinus floor elevation can be performed, and when RBH is <6 mm, the lateral approach is usually recommended [4].

Maxillary sinus augmentation encompasses various grafting biomaterials which can regenerate hard tissues, increasing bone volume and allowing for implant placement [5,6]. As an alternative to autogenous bone graft (AB), bone substitutes such as allografts (AG), xenografts (XG), alloplasts (AP), and bioactive agents (Bio) can be used as single material or combined among them or with autogenous bone, to achieve effective regeneration.

It was hypothesized that new bone formation (NBF%) into the graft may depend on local factors such as anatomical features and dimensions of the maxillary sinus, Schneiderian membrane thickness, distance from the sinus floor, and also RBH [7,8,9,10,11]. A recent well-conducted meta-analysis suggested that NBF may increase by approximately 2% per each mm of increase in residual bone height [12].

A meta-regression review by Chao et al., published in 2010, aimed to identify the influence of initial bone height on implant survival either through lateral window or osteotome technique [13]. The review included 12 studies related to lateral window technique and pooled 406 patients and 1644 implants for analysis. The review concluded that implant survival rate increases in a positive trend when the initial bone height increases from approximately 1 to 5 mm. Implant survival achieved a plateau at a high level when the initial bone height was >5 mm [13]. A study by Stacchi et al., published in 2018, reported that narrower sinuses were found to induce more effective new bone formation than larger sinuses [8]. It was also reported that sinus bone walls, as well as the Schneiderian membrane, are rich in osteoprogenitor cells and have a significant influence in new bone formation. Histomorphometric evaluation confirmed an inverse relationship, i.e., the bone formation decreases as the distance from the sinus wall increases. The bone formation in the sinus starts from the sinus wall and gradually approaches the apex of the implants [11]. The impact of RBH on new bone formation has been investigated through clinical studies and meta-analyses [9,10,11,12,13]. However, no comprehensive review evaluated a possible combined effect of RBH and the grafting material in promoting the formation of new bone through a network meta-analytic approach. Network meta-analysis is a statistical tool that allows the simultaneous comparison of multiple treatments, as opposed to traditional meta-analysis, which only allows pairwise comparisons [14,15,16]. The objective of the present study was to investigate, through a network meta-analysis, the effect of RBH and grafting material on new bone formation after lateral sinus augmentation using different biomaterials. The null hypothesis is that new bone formation is independent of RBH and of the grafting material used. The alternative hypothesis is that, in order to achieve the highest NBF, the choice of the grafting material may depend upon RBH.

## 2. Materials and Methods

### 2.1. Design and Registration

The protocol for systematic review and network meta-analysis (NMA) on the effect of RBH on new bone formation after lateral sinus augmentation using different biomaterials was registered on PROSPERO. The protocol registration ID was CRD42022331993. We followed the Preferred Reporting Items for Systematic Reviews and Meta-analysis (PRISMA) statements for reporting this systematic review and meta-analysis.

### 2.2. Search Strategy and Selection Criteria

PUBMED, SCOPUS, Cochrane Central, and Web of Science databases were used to identify relevant randomised controlled clinical trials (RCTs) until 31 December 2022. There were no limitations on the year of publication and publication language.

All RCTs (both split-mouth and parallel studies) that involved the test and control groups were considered. Each study should have at least one biomaterial for the test group and at least one placebo or biomaterial for the control group. The exclusion criteria were: (1) narrative reviews, letters, personal opinions, book chapters, case reports, conference abstracts, and meetings; (2) duplicate publications; (3) experimental in vitro and in vivo animal studies; (4) studies using the same biomaterial in both the test and control groups.

The PICO framework of the present review was as follows. We included participants (P) requiring sinus lift procedures irrespective of residual bone height. There were no age or gender limitations. Intervention group (I): Sinus lift procedure with the use of at least one biomaterial. Control group (C): Sinus lift procedure with self-healing/no material or with any materials other than those used in the intervention group. The primary outcome measure (O) was new bone formation determined histomorphometrically (%) after the sinus lift procedure with/without grafting biomaterial. The secondary outcome measure was the incidence of any adverse events or complications. The duration of the healing period at the time of bone biopsy had to be no less than 2 months.

A literature search was undertaken using electronic databases such as PUBMED, SCOPUS, Web of Science, and Cochrane Central. The key words used were sinus lift, sinus lift procedure, maxillary sinus lift, residual bone height, RBH, new bone formation, histomorphometry, bone histomorphometry, histomorphometric analysis, and lateral technique. The Boolean search strategy was ((((((maxillary sinus) OR (sinus lift)) OR (maxillary sinus lift)) OR (maxillary sinus lift technique)) OR (maxillary sinus lift lateral)) OR (maxillary sinus augmentation)) OR (sinus lift)) OR (sinus lift procedure)) AND (((histomorphometric) OR (histomorphometric analysis)) OR (bone histomorphometry)). The last electronic search was conducted on 9 January 2023. The reference lists of all identified RCTs and relevant systematic reviews were scanned for possible additional studies. A hand search was also performed on the main journals of oral and maxillofacial surgery and implant dentistry.

Two reviewers independently screened the titles and the abstracts of the retrieved articles to determine all the eligible studies that met the inclusion criteria. The differences in agreement between examiners was assessed using Cohen’s kappa test. When the abstract was not available or was not sufficient to allow unequivocal evaluation, the full text was obtained. The published papers that were not eligible were excluded. Disagreements between the two authors were discussed until a consensus was reached. The full text of all the eligible articles was obtained. The same two reviewers assessed the features of each study to confirm inclusion for data analysis or to exclude the study. The reasons for exclusion at this stage were noted. In case of disagreement between the reviewers, a consensus was achieved by consulting with a third reviewer.

### 2.3. Data Collection

The data related to authors, year, sponsorship, number of patients included and assessed in each group, age, gender, smoking, habits, the type of sinus lift technique, residual bone height, type of biomaterial used in the test and control groups, including no biomaterial/placebo, use of a covering membrane, number of sinus lifts performed, number of dental implants inserted, length of follow-up, new bone formation as a percentage, adverse events, complications, and the conclusions of each included article were extracted by one reviewer. The other reviewed critically and validated the appropriateness of the data. To assess the effect of RBH, the data were split according to the mean RBH value, considering the value of 4 mm as a threshold, and separate network meta-analyses were performed for data obtained from sinuses with RBH < 4 mm and RBH ≥ 4 mm.

### 2.4. Outcome Variables

The outcome variables were new bone formation as a percentage and, if available, residual biomaterial % and connective tissue %. Adverse events, biological complications (e.g., fistulae, sinus infection, peri-implantitis, peri-implant mucositis), mean values and standard deviations (SD) for primary outcomes, and number of sinus lift procedures (*n*) were extracted or, when possible, estimated. When an article did not provide the mean values and standard deviations, or when data were missing, the corresponding author was contacted in order to provide missing information. In the case of no or an unsatisfactory reply, the study was excluded.

### 2.5. Data Analysis for Network Meta-Analysis

Network meta-analysis was performed using metan commands in STATA v17.0. A series of graphs and plots were generated to demonstrate the network connections between interventions. They were illustrated in nodes and edges. Nodes denote the competing treatments, while the edges represent the available direct comparisons between pairs of treatments [14]. The network plots use weighting and colouring schemes and reveal important differences in the characteristics of treatments or comparisons. These differences may indicate a potential violation underlying network meta-analysis [15,16]. The contribution plot estimates the contribution of direct comparisons in network estimates. The plot helps to identify the large or small contributions that enhance the understanding of evidence flow.

Consistency is a key for network meta-analysis, which is indicated by a closed loop formed by three or more treatments and direct and indirect estimates do not differ substantially. Loops in which the lower confidence interval limit of the inconsistency factor does not reach the zero line are considered to present statistically significant inconsistency. Predictive interval plots (Prl) were generated to identify the most effective material that could perform best in future clinical studies. The surface under the cumulative ranking curves (SUCRA) was ranked using probabilities. The relative ranking of treatments (dissimilarity) was ranked using multidimensional scale (MDS).

### 2.6. Risk of Bias

The methodological quality of the included studies was independently evaluated by two reviewers as part of the data extraction process. The risk of bias of the included trials was assessed based on the following criteria: randomisation method, concealed allocation of treatment, blinding of outcome assessors, completeness of outcome assessment reporting, completeness of information on reasons for withdrawal by trial group, other biases (sample size calculation, definition of inclusion/exclusion criteria, and comparability of control and test groups at entry). All such criteria were scored as adequate/inadequate/unclear. The blinding of participants and personnel (performance bias) was not considered, because in sinus lift procedures, neither the surgeon nor the patient can be efficiently masked to the bone graft material used, especially if it is autogenous bone.

Studies were classified as follows: low risk of bias (plausible bias unlikely to seriously alter results) if all criteria were judged adequate; moderate risk of bias (plausible bias that raises some doubt about the results) if one or more criteria were considered unclear; or high risk of bias (plausible bias that seriously weakens confidence in the results) if one or more criteria were judged inadequate. The criteria for assessing the risk of bias of RCTs were adapted from the tool reported in the Cochrane Handbook for Systematic Reviews of Interventions. Disagreement between the two reviewers was resolved by consulting with a third reviewer. Publication bias for the main comparisons was assessed using a funnel plot.

### 2.7. Heterogeneity

To assess the impact of heterogeneity in the meta-analysis, Higgins’s I^2^ test was used. This statistic represents the proportion of variability that is due to heterogeneity rather than to sampling error. According to the I^2^ statistical test, the heterogeneity could be low (I^2^ < 50%) or high (I^2^ > 50%). If heterogeneity was high, the possible sources of heterogeneity were explored using Moses–Shapiro–Littenberg regression and subgroup analyses. Publication bias was investigated using Deek’s funnel plot asymmetry test. All statistical tests were two-sided. A *p*-value less than 0.05 will be considered statistically significant.

The quality of evidence was assessed based on the GRADE approach [17].

## 3. Results

**Figure 1:**
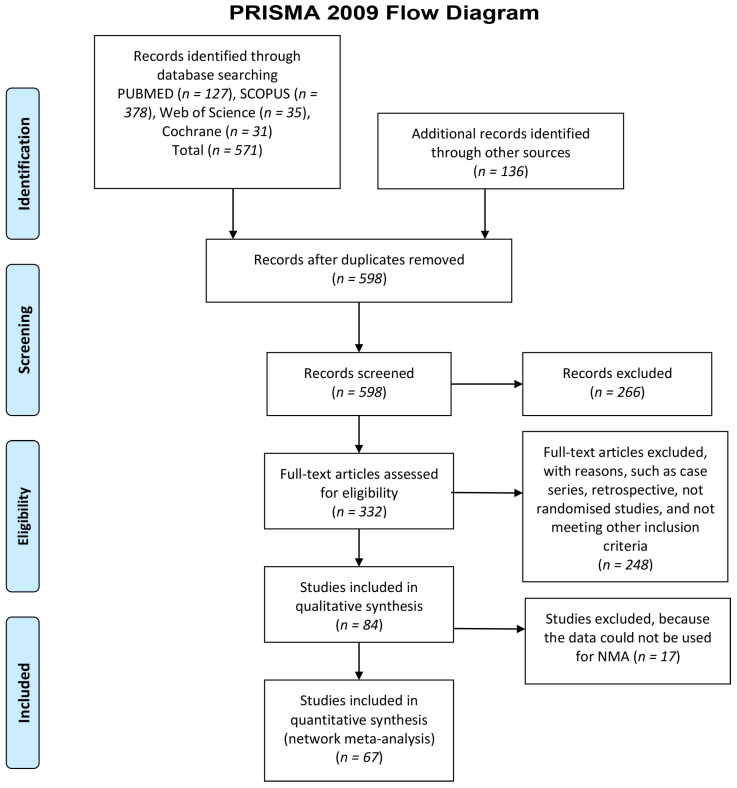
Study flow diagram showing the study selection process.

The flow of the study selection procedure is illustrated in Figure 1.

The search strategy identified 707 articles from the databases, including additional records identified through other sources. After the duplicates were removed (N = 109), a total of 598 articles was included for further screening through the titles and abstract. In total, 266 records did not meet the inclusion criteria based on titles and abstract were excluded. Upon full text assessment by two authors, 84 studies were selected for data extraction for qualitative analysis [18,19,20,21,22,23,24,25,26,27,28,29,30,31,32,33,34,35,36,37,38,39,40,41,42,43,44,45,46,47,48,49,50,51,52,53,54,55,56,57,58,59,60,61,62,63,64,65,66,67,68,69,70,71,72,73,74,75,76,77,78,79,80,81,82,83,84,85,86,87,88,89,90,91,92,93,94,95,96,97,98,99,100,101]. A further 17 studies were not considered for quantitative analysis because of the following reasons (some studies were excluded for multiple reasons): (a) the same biomaterial group was used in the test and control group [20,25,26,27,31,32,39,42,49,53,55,73,90], for example, 2 studies investigated the effect of using or not using phototherapy [20,39], another study compared biopsies collected from antrostomy to those collected crestally from the same patients, grafted with porcine bone [31], another compared monophasic vs. biphasic alloplastic materials (both belonging to our category “AP”) [53], and another compared biopsies of autogenous graft harvested at different healing times [55]; (b) 4 studies did not report RBH, and could not be categorized in the NMA [52,53,54,57]; (c) 3 studies were not randomised [20,52,54]; (d) 1 study investigated the vertical course of bone regeneration and did not provide separate results for different materials [21]. Therefore, 67 studies were eligible for quantitative analysis and were feasible to conduct network meta-analysis. Cohen’s kappa values for inter-reviewer agreement for title/abstract and full-text articles selection were 0.92 and 0.93, respectively, indicating almost perfect agreement. A total of 1955 patients were included in the selected studies, with 2405 sinus lift procedures performed. The characteristics of the included studies are reported in Table 1. In this table, the different materials are mostly reported as in the original article.

The graft materials used were autografts (Auto), xenografts (XG) (bovine bone, equine bone, porcine bone, with/without the addition of collagen), allografts (AG), alloplasts (AP) (bioactive glass, hydroxyapatite, beta-tricalcium phosphate, polylactic-co-glycolic acid), bioactive agents (Bio) (recombinant human bone morphogenetic protein-2 (rhBMP-2), recombinant human growth differentiation factor (rhGDF-5), mesenchymal stem cells, enamel matrix derivative, autologous platelet concentrates), hyaluronic acid (HA), and a combination of two or more materials. The time at which biopsy was performed averaged 6.6 ± 2.6 months (range 2 to 15 months) after grafting. The most frequent healing time among the included studies was 6 months, which was used in 52 studies.

Network meta-analysis could only be performed for new bone formation, as histomorphometric data on residual biomaterial and connective tissue were rarely provided in the included studies.

**Figure 2:**
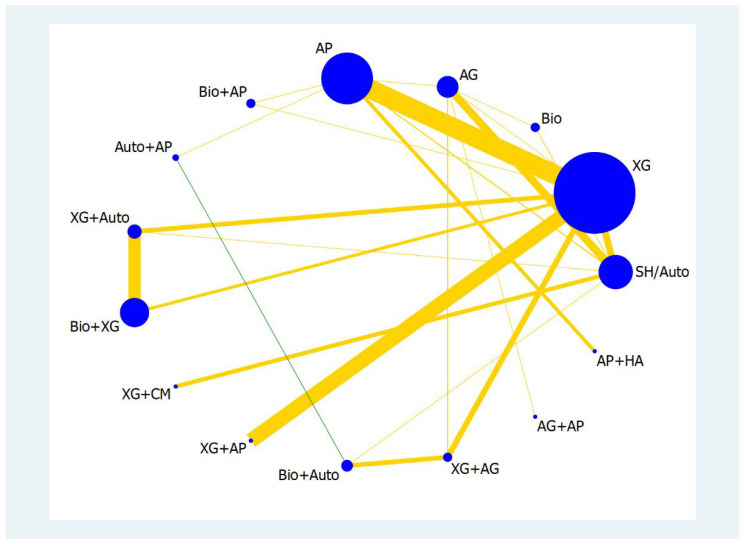
Network plot for <4 mm RBH. The size of blue circles (nodes) is proportional to the number of patients for the specific group of graft material. The thickness of lines between nodes is proportional to the number of comparisons between two or more groups of graft material. The colour of the lines indicates the risk of bias: yellow represents moderate and green indicates low risk of bias.

**Figure 3:**
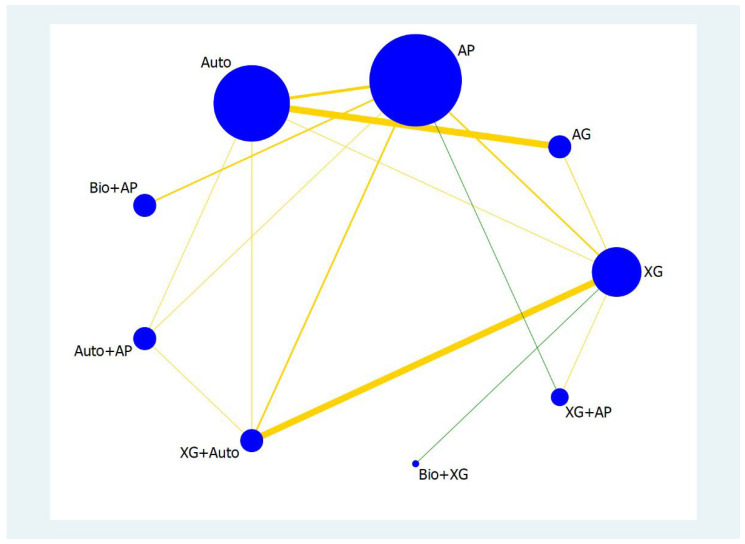
Network plot for the ≥4 mm RBH. The meaning of the circle and line size is as in Figure 2. The yellow colour indicates a moderate risk of bias and the green colour indicates a low risk of bias.

A network geometry plot illustrates the most common comparison between biomaterials. The nodes represent the number of samples obtained from different studies for a specific biomaterial and the thickness of the line represents the number of comparisons. The more the comparisons, the thicker the line between the two biomaterials (Figure 2 and Figure 3).

The number of samples was higher for XG, AP, auto, and Bio+XG for <4 mm RBH outcome and the most frequent comparisons were between XG and AP; XG and XG+AP; AG and Auto. Similarly, the most common comparison was between Bio+AP; XG and Auto+AP for ≥4 mm RBH outcome. Although they are the most frequently compared biomaterials in the sinus augmentation procedures, their effect sizes vary and hence effectiveness differs. IF, Prls (predictive intervals), and SUCRA ranking should be considered before making informed decision and assessing the quality of evidence existing among biomaterials.

Loops in which the lower confidence interval (LCI) limit of the inconsistency factor (IF) does not reach the zero line are considered to present statistically significant inconsistency. Therefore, all loops present within <4 mm RBH have a lower confidence interval value zero and therefore the IF is not significant within these comparisons. However, when IF was seen in the entire loop, such as in the XG, AP, and Bio+AP group, there was statistically significant inconsistency.

According to the predictive intervals (Prls), XG+AG and XG+AP for <4 mm RBH and Auto and Bio+XG for ≥4 mm RBH were predicted to be the best combination biomaterials that are most likely to perform better in future clinical studies.

The treatment effect of biomaterials considered for RBH < 4 mm was 8.97 (CI 95%: −3.60, 21.56) and 1.93 (5.88, −9.59) for XG+AG and XG+AP, respectively. This means that the combination of xenografts and allografts ranked better and performed better than the other biomaterials in the percentage of new bone formation. XG+AG performed 8.97 times better compared to other biomaterials.

**Figure 4:**
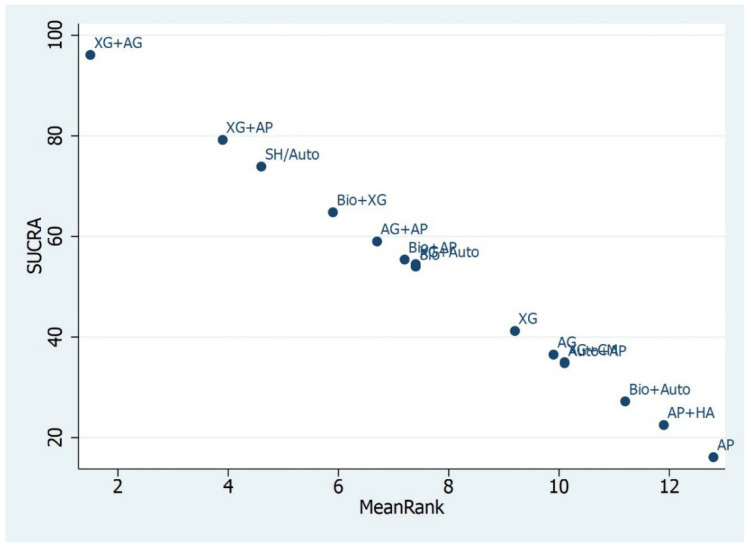
SUCRA Ranking for <4 mm RBH. XG+AG biomaterial was ranked highest among all the biomaterials compared and performed best in terms of clinical outcome. Alloplast performed worst when compared with other biomaterials in terms of clinical outcomes.

**Figure 5:**
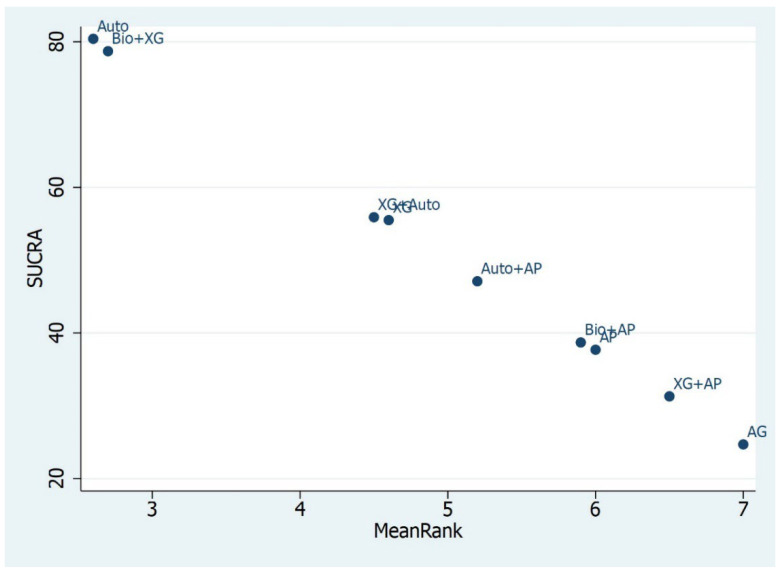
SUCRA Ranking for ≥4 mm RBH. Auto and Bio+XG ranked first, AG ranked last.

Similarly, for RBH ≥ 4 mm, the treatment for Auto, Bio+XG, and XG+Auto was 9.86 (12.71, −15.06), 2.01 (7.74, −13.17), and 1.86 (6.88, −11.64), respectively. In this case, autologous graft, bio+XG, and xenografts combined with autogenous graft ranked best among other biomaterials and performed best in the percentage of new bone formation. According to the SUCRA, the highest-ranked biomaterials for the <4 mm RBH group were XG+AG, XG+AP, and SH/Auto, with the alloplasts alone in last position. Similarly, the SUCRA ranking for the biomaterials for ≥4 mm RBH were Auto, Bio+XG, and XG+Auto, with allografts alone as the last one (Figure 4 and Figure 5).

### Quality of Evidence

**Figure 6:**
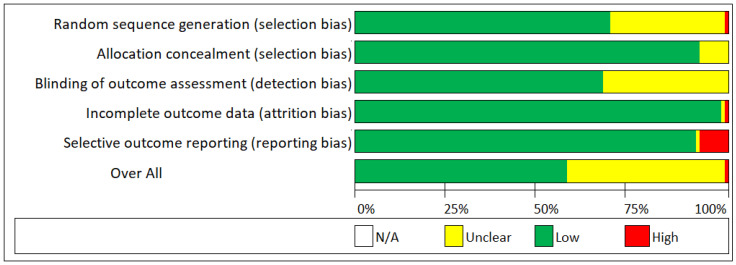
Risk of bias summary.

The quality of evidence was low for all the biomaterials included in this review. This is due to wider 95% confidence intervals for direct, indirect, and network evidence. In order to have a moderate and high level of evidence, the 95% CI should have been narrower. Figure 6 shows the risk of bias assessment results for the included studies.

## 4. Discussion

The volume and linear dimension of the maxillary sinus cavity gradually tends to increase upon tooth loss and ageing, due to sinus pneumatization, parallel to a progressive decrease of the dimension of the residual crest [102]. This leads to the atrophy of the alveolar process in the posterior maxilla. The maxillary sinus augmentation procedure has led an innovative approach in managing the atrophic alveolar process in the maxillary sinus region. The technique underwent numerous modifications of the original protocols, with the introduction of a number of materials, implant types, and surgical approaches to the sinus. In general, over the years, maxillary sinus augmentation has proved to be a predictable technique, whose clinical outcomes can be affected by a number of factors, with the anatomical features at the time of surgery among the most investigated. A reduced dimension of the crest in the sinus region may decrease the regenerative potential of the sinus floor, and also implies a reduced distance from the posterior alveolar artery (PSA) to the maxillary sinus floor and alveolar crest. Therefore, the risk of injuring the PSA during sinus augmentation procedure increases, which may complicate the surgical technique [102]. Previous studies have suggested that an increase of RBH may have direct benefits for implant survival compared to sinuses with low RBH [103,104]. It is also believed that there is an influence of the Schneiderian membrane on new bone formation [105]. The objective of the present network meta-analysis was to investigate if RBH has an effect on new bone formation after lateral sinus augmentation, taking into account the use of different biomaterials. The advantage of using such a statistical approach is that different biomaterials can be compared amongst each other, even though there was no study performing a direct comparison for some of them.

Our results showed that for RBH of <4 mm, XG+AG biomaterial ranked best for successful bone regeneration, and for RBH ≥4 mm, Auto, followed by Bio+XG and XG+Auto biomaterials, ranked best. It is known that autogenous bone, allografts, and bioactive agents all have osteogenic and/or osteoinductive properties. This confirms that to ensure a predictable bone formation, it is preferable to associate an osteogenic/osteoinductive component to an osteoconductive scaffold. According to the SUCRA rankings in the network meta-analysis, XG+AG (<4 mm) and Auto (≥4 mm) resulted in the two superior specific bone substitutes in terms of new bone formation after sinus augmentation procedures at different levels of RBH. A recent study by Stacchi et al. demonstrated that a percentage of mineralised tissue formation occurs at different rates in different anatomical locations within the same maxillary sinus and also illustrated a negative correlation between sinus width and new bone formation. In their study, RBH did not influence new bone formation [104].

The inconsistency factor (IF) in our NMA was represented by the loops formed between direct and indirect comparison between biomaterials [106]. There was no loop formed for the XG+AG and XG+Auto biomaterials; hence, there was no statistically significant IF. It demonstrates that there were no statistical differences in the effect sizes between clinical studies involving different biomaterials (especially in XG+AG and Auto).

Predictive intervals (Prls) provide information in the form of the range in which future studies are predicted to lie [107,108]. They can also help in giving information on heterogeneity and evade issues that arise due to the I^2^ statistic. According to the NMA, XG+AG, and XG+AP for <4 mm RBH, and Auto and Bio+XG for ≥4 mm RBH, these are the combinations that most probably will perform better in future clinical investigations. Predictive intervals should be used in clinical settings when deciding the choice of biomaterial and they recommend the most optimal way of approach in sinus augmentation.

The limitations of SUCRA rankings should be considered given they vary due to a number of factors, including the number of multiple outcomes, the cost of biomaterials and clinicians’ familiarity about handling the biomaterials, the process of calculating the rankings, and the apparent differences between the treatments. Another limitation of the study was the variable healing time before performing the biopsy, ranging from 2 to 15 months. In order to avoid excessive data fragmentation, it was decided not to further split the data into different healing times.

## 5. Conclusions

Different biomaterials performed differently according to RBH after sinus augmentation. The combination of xenograft and autograft ranked best in performance for <4 mm RBH, while autogenous bone and the combination between bioactive agents and xenograft ranked best when RBH was ≥4 mm. These biomaterials are also most likely to perform best in future clinical studies. In order to achieve a greater amount of new bone formation, the amount of residual bone may be critical in determining the choice of material.

## Figures and Tables

**Table 1:** Characteristics of the included studies.

1st Author, Year	No. of Patients	No. of Sinus Lifts	Age, Years Mean ± SD (Range)	Residual Bone Height (mm)	Months (Follow-Up)	CTR Material	Test Material/Technique
Mendes, 2022 [18]	30	30	(50–70)	<5	6	Autogenous bone	G2: β-TCP ChronOS; G3: Beta-TCP
Harlos, 2022 [19]	36	36	53.8	<3	8	DBBM+Autogenous bone	G2: Auto+PRF; G3: XG
Arshad, 2021 [20] *	44	44	40.5 ± 8.5	۔	1	AG	Allograft (+LED Group)
Beck, 2021 [21] *	55	85	51.82 ± 9.93	4.58 ± 2.5	6	DBBM	XG+auto; XG+bio
Zahedpasha, 2021 [22]	10	20	45.65 ± 5.74 (39–51)	G1:4.88 ± 1.63; G2:5.36 ± 1.55	6	Self-healing (no graft)	Bovine bone (Cerabone)
Trimmel, 2021 [23]	26	30	57.93 ± 7.79 (test); 55.33 ± 8.55 (ctr)	2.93 ± 1.14 (test); 3.48 ± 1.04 (ctr)	3 (test) 6 (ctr)	A-PRF	Albumin-coated bone allograft (SACBA)
Correia, 2021 [24]	12	24	59.7 ± 8.7	3.20 ± 0.93	6	Autologous bone	Porcine bone
Chaushu, 2020 [25] *	29	38	55.5 ± 10 (39–74)	<3	9	Allograft particles	Allograft block
da Silva, 2020 [26] *	13	30	55 ± 8.1	3.11 ± 0.83 (ctr); 2.38 ± 0.75 (test)	6	DBBM	XG (granules 1–2 mm) (Lumina porous)
Grasso, 2020 [27] *	16	23	54 ± 7	<4	6	Deproteinized equine bone mineral (DEBM)	Anorganic bovine bone (DBBM)
Kim, 2020 [28]	37	51	53.0 ± 8.17; 51.07 ± 9.67; 54.15 ± 8.24	<5	6	Anorganic bovine bone	Mineralized cancellous bone allograft
Velasco-Ortega, 2020 [29]	24	24	BCP: 57.63 ± 13.97; BCP+HA: 60.63 ± 11.21; ABBM: 49.5 ± 11.28	<3	9	Demineralized bovine bone	Test 1: TCP (particle size 250 to 1000 μm); Test 2: TCP as in test 1 + crosslinked HA 2:1
Pereira, 2020 [30]	40	40	32–65	<5	6	Autologous bone (G1)	G2: Bioactive glass; G3: Bioactive glass +Autologous bone; G4: Bio-Oss; G5: Bio-Oss+Auto
Tanaka, 2019 [31] *	12	12	55.3 ± 11.7	<4	9	Collagenated corticocancellous porcine bone (Alveolar Crest Sites)	Collagenated corticocancellous porcine bone (Antrostomy sites)
Pang, 2019 [32] *	25	28	56.67 ± 10.53	2.92 ± 2.17 (Inducera)/3.69 ± 4.85	6	DBBM (Bio-Oss)	Calcium phosphate crystal double-coated bovine bone
Batas, 2019 [33]	6	12	۔	<3	6	DBBM	DBBM+PRGF
Oh, 2019 [34]	56	60	54.3 (20–69)	2–6	6	DBBM	Biphasic calcium phosphate
Scarano, 2018 [35]	23	27	52	NR	6	Group 1: Collagen porcine bone + CM	Autologous bone
Nizam, 2018 [36]	13	26	49.92 ± 10.37	<5	6	DBBM+L-PRF	DBBM
Taschieri, 2016 [37]	20	20	49–69	<4	6	DBBM	BCP+PRP
Menezes, 2018 [38]	21	27	NR	<5	6	Autogenous bone graft	Biogran (AP) + Autologous bone
Theodoro, 2018 [39] *	12	12	48.12 ± 6.24	4 to 5	6	AB/HA	AB/HA+LLLT
Pareira, 2017 [40]	22	36	NR	<5	6	Autogenous bone	Test 1: Auto; Test 2: Auto+Biogran
Stacchi, 2017 [41]	28	52	60.1	2	6	ABB	NHA
Lee, 2017 [42] *	16	20	44.04 ± 4.48	Ctr: (2.06 ± 0.43 mm)/Test: (1.90 ± 0.80 mm)	6	XG (DBBM, ctr)	XG (DPBM, test)
Rodriguez y Baena, 2017 [43]	8	12	56 ± 13	<4	6	Deproteinized bovine bone	Poly(lactic-co-glycolic acid/Hydroxyapatite
Comert Kiliç, 2017 [44]	26	18	31.51 ± 8.52 (ctr); 34.01 ± 9.59 (test)	<7	6	β-TCP	β-TCP+PRP
Dogan, 2017 [45]	13	26	(33–69)	<4	4	Collagenated heterologous bone graft	Hyaluronic matrix and collagenated heterologous bone graft
Kolerman, 2017 [46]	13	26	58	<5	9	BCP	Freeze dried bone allografts
Meimandi, 2017 [47]	10	20	(30–60)	2 to 4	6	Alloplast	Bone graft + Growth factors
Portelli, 2017 [48]	8	12	56	4 to 5	8	Xenografts	Alloplast
Meymandi, 2017 [49] *	9	18	(42–57)	12 to 13	6	Easy Graft Crystal (Alloplast)	Nano Bone (Alloplast)
Nery, 2017 [50]	10	20	(35–75)	3 and 5	6	β-TCP/HA (BC)	β-TCP/HA mixed with EMD (BC+EMD)
Pereira, 2017 [51]	30	30	NR	<5	6	Biogran	Biogran with autogenous bone graft and Autogenous bone graft
Amoian, 2016 [52] *	20	20	49 ± 4.32	NR	6	DFDBA	DFDBA
Jelusic, 2016 [53] *	60	67	55.92	NR	6	Monophasic (100% ß-TCP)	Biphasic (60% HA and 40% ß-TCP)
Nappe CE 2016 [54] *	18	25	67	NR	6	XG	Alloplast + Allograft
Duque Netto, 2016 [55] *	10	20	NR	<4	2 and 6	Auto 6 months	Auto 2 months
Ahmet, 2016 [56]	20	20	53.8 (47–65)	<5	5	Biphasic CS + Alloplast (60%HA, 40% β-TCP)	Biphasic CS + DBBM
Badr, 2016 [57] *	22	22	36 (17–73)	NR	6	Autograft	Auto+PRP
Kim, 2016 [58]	30	30	54.6 ± 0.42	2.50 ± 1.01/2.87 ± 0.74	6	Auto+PC	AG+XG+PC
Alayan, 2016 [59]	16	40	57.7 ± 0.43 (ctr);54.6 ± 0.33 (test)	<5 and >1	5	Anorganic bovine bone + Autogenous bone	Collagen-stabilized anorganic bovine bone
Danesh-Sani, 2016 [60]	10	20	(25–72)	<5	6 to 8	Autogenous bone	BCP (60% hydroxyapatite and 40% β-TCP)
de Oliveira, 2016 [61]	15	21		2,2	6	Bovine bone	Bovine+BMC (bone marrow concentrate)
Payer, 2015 [62]	6	12	58.2	<3	6	Bovine bone	Bovine bone + Tibial BM aspirate
Kim, 2015 [63]	41	41	52.37	<3	6	Xenografts	rhBMP-2 + Microporous BCP
Kim, 2015 [64]	127	127	53.19 (test); 53.15 (ctr)	<4	3	Xenografts	rhBMP-2 + Microporous BCP
Sehn, 2015 [65]	29	34	51.32 ± 6.44	<5	6	Fresh-frozen bone allograft	Bovine bone mineral + Fresh-frozen bone allograft
Taschieri, 2015 [66]	6	12	(48–71)	<4	6	Xenografts	Alloplast
Xavier, 2015 [67]	15	30		6	<3	Autogenous	Allograft
Pasquali, 2015 [68]	8	16	55.4 ± 9.2	<4	6	Bio-Oss	BMAC
de Lange, 2014 [69]	5	10	66 (64–71)	2,4	12	DBA	BCP (Straumann BoneCeramic; Institut Straumann AG)
Correia, 2014 [70]	6	12	(42–64)	2–4.6	6	Autogenous bone	Xenograft
Garlini, 2014 [71]	5	10	57	<5	6 to 8	Xenograft	Algipore
Wildburger, 2014 [72]	7	14	58 (47–72)	<3	3 and 6	Bovine bone	BOVINE Bone + MSC
Torres, 2013 [73]	93	13	<65:38; >65:55	<7	6	DBBM + membrane	DBBM
Froum, 2013 [74]	24	48	61.2 ± 7.7	4 to 5	6 to 9	Allografts	Bone grafts + bioactive protein
Froum, 2013 [75]	24	24	61.2	4 to 5	4–5 and 7–9	Xenograft	XG+PDGF
Khairy, 2013 [76]	15	10	38 (22–54)	<5	6/4 and 6	Autogenous bone	Autologous bone + PRP
Schmitt, 2013 [77]	30	36	(38–79)	<4	5	Autologous bone	Mineralized cancellous bone Allograft
Tosta, 2013 [78]	30	30	(18–70)	3 and 6	9	Autogenous	BCP
Anitua, 2012 [79]	5	10	52 ± 11 (29–73)	1–3	5	DBBM	DBBM+PRGF
Kao, 2012 [80]	22	20	50.8	<5	6	Bio-Oss	Bio-Oss + rhBMP-2/ACS
Kurkcu, 2012 [81]	23	23	48.65	<5	6,5	Xenografts	Alloplast
Lindgren, 2012 [82]	11	22	67 (50–79)	<5	36	Xenografts	Alloplast
Zhang, 2012 [83]	10	11	43.5 (test);46.2 (ctr)	6	<5	Xenografts	Bone Grafts and Growth Factors
Wagner, 2012 [84]	85	117	52.5 (22.7–82.6)	2 to 5	6	Biphasic Ca(PO)_4_ + Fibrin sealant	Autogenous bone graft with Bovine Xenograft
Pikdöken, 2011 [85]	24	24	59.83 (57.92)	4	<5	Xenografts	Autogenous + XG
Stavropoulos, 2011 [86]	31	31	53.8 ± 12.1	<5	4	rhGDF-5/b-TCP/3-month	Biologics
Rickert, 2011 [87]	23	22	60.8 ± 5.9	1 to 3	4	Bovine bone mineral + Autogenous bone	Bovine bone mineral + Autogenous stem cells
Sauerbier, 2011 [88]	36	44	56.6	2 to 3	3 to 4	Autogenous + Xenograft	Bone grafts + mesenchymal cells
Galindo-Moreno, 2011 [89]	28	28	47.3 ± 9.8	<5	6	Bovine+AB 1:1	Bovine + AB 4:1
de Vicente, 2010 [90] *	35	42	(34–69)	<4 (severely atrophic)	9	Bovine-derived hydroxyapatite (2-stage)	Bovine-derived hydroxyapatite (1-stage)
Felice, 2009 [91]	10	20	50 (35–60)	1–5	6	DBBM	No graft + rigid synthetic resorbable membrane
Cordaro, 2008 [92]	37	48	NR	≥3 and <8 mm	8	Straumann Bone Ceramic	Anorganic bovine bone
Froum, 2008 [93]	12	21	NR	<5	6 to 8	Xenograft	Alloplast
Galindo-Moreno, 2008 [94]	5	10	62 (45–78)	<5	6	Bovine+AB	Bioglass + AB
Froum, 2006 [95]	13	22	59	<5	8	Mineralized cancellous bone allograft	Anorganic bovine bone
Zijderveld, 2005 [96]	10	16	(18–70)	5 ± 2.05	12	Autologous chin bone	β-TCP
Raghoebar, 2005 [97]	5	10	58.4 ± 1.9	<5	3	Autogenous bone	Autogenous bone + PRP
Szabo, 2005 [98]	20	40	52	<5	6	Autogenous	Alloplast
Zerbo, 2004 [99]	9	14	52	6	<4	Autogenous bone	TCP
Wiltfang, 2003 [100]	35	35	45 (37–54) (test); 47 (32–64) (ctr)	2 to 7	6	B-TCP	B-TCP + PRP
Hallman, 2002 [101]	21	22	54	<5	12 to 15	Autogenous bone	Autogenous + XG

* = studies not included in the network meta-analysis; SD = standard deviation; CTR = control; DBBM = deproteinized bovine bone material; TCP = tricalcium phosphate; PRF = platelet-rich fibrin; PRP = platelet-rich plasma; PRGF = plasma rich in growth factors; HA = hydroxyapatite; AG = allograft; XG = xenograft; NHA = nano-hydroxyapatite; BC = Bone Ceramic; EMD = Emdogain (enamel matrix derivative); DFDBA = demineralized freeze-dried bone allograft; BMP = bone morphogenetic protein; BCP = biphasic calcium phosphate; BMAC = bone marrow aspirate concentrate; MSC = mesenchymal stem cell; PDGF = platelet-derived growth factor; GDF = growth/differentiation factor; ACS = absorbable collagen sponge.

## Data Availability

The authors are available to share the data upon request.
